# An atypical immunophenotype in generalized eruptive histiocytosis: a case report of coexisting scattered S-100 positivity and seropositivity for Anti-nRNP/Sm antibodies

**DOI:** 10.3389/fimmu.2026.1791255

**Published:** 2026-08-18

**Authors:** Xinran Du, Siyu Pu, Ruizheng Zhu, Dongjie Guo

**Affiliations:** Department of Dermatology, Yueyang Hospital of Integrated Traditional Chinese and Western Medicine, Shanghai University of Traditional Chinese Medicine, Shanghai, China

**Keywords:** autoimmune serology, case report, CD68, generalized eruptive histiocytosis, S-100

## Abstract

Generalized eruptive histiocytosis (GEH) is a rare non-Langerhans cell histiocytosis characterized by multiple symmetrical papular eruptions. We describe an atypical presentation of GEH in a 60-year-old man with scattered S-100 immunopositivity and seropositivity for anti-nRNP/Sm antibodies. Histopathology revealed dense perivascular and interstitial infiltrates of histiocytes, plasma cells, and occasional eosinophils within the upper to mid-dermis, while immunohistochemistry revealed diffuse CD68 positivity, CD1a negativity, and scattered S-100 positivity. The unusual immunophenotype and concurrent abnormal autoimmune serology results suggest a possible association between local histiocytic activation and systemic immune dysregulation. To the best of our knowledge, no previous reports have linked GEH with seropositivity for anti-nRNP/Sm antibodies. The coexistence of this serological finding with an atypical S-100+ immunophenotype in our case provides further insight into the immunopathological features of GEH.

## Introduction

Histiocytoses are rare disorders characterized by abnormal accumulation of cells derived from macrophages, dendritic cells, or monocytes in various tissues and organs. In 2016, the Histiocyte Society introduced an updated classification that categorized these disorders into five groups: (1) Langerhans-related histiocytoses, (2) cutaneous and mucocutaneous histiocytosis, (3) malignant histiocytosis; (4) Rosai–Dorfman disease, and (5) hemophagocytic lymphohistiocytosis and macrophage activation syndrome (1). Generalized eruptive histiocytosis (GEH) is classified within the second category. It was first described by Winkelmann and Müller in 1963 (2), who reported characteristic features including widespread symmetric papular eruptions on the trunk and proximal extremities, minimal mucosal involvement, progressive development, and spontaneous regression, often leading to hyperpigmentation. GEH typically occurs in adults aged 30–60 years, with fewer pediatric cases reported (3). Here, we present an adult male patient with an atypical immunophenotype of GEH, characterized by scattered S-100 positivity and concurrent seropositivity for anti-nRNP/Sm antibodies, suggesting possible immunologic interplay.

## Case description

A 60-year-old male presented with multiple asymptomatic red papules on the medial aspects of both thighs, which had developed insidiously without an identifiable trigger two years prior. Over time, similar lesions appeared on the trunk and the medial aspects of both upper arms, prompting him to seek medical attention at our hospital on October 5, 2024.

**Figure 1:**
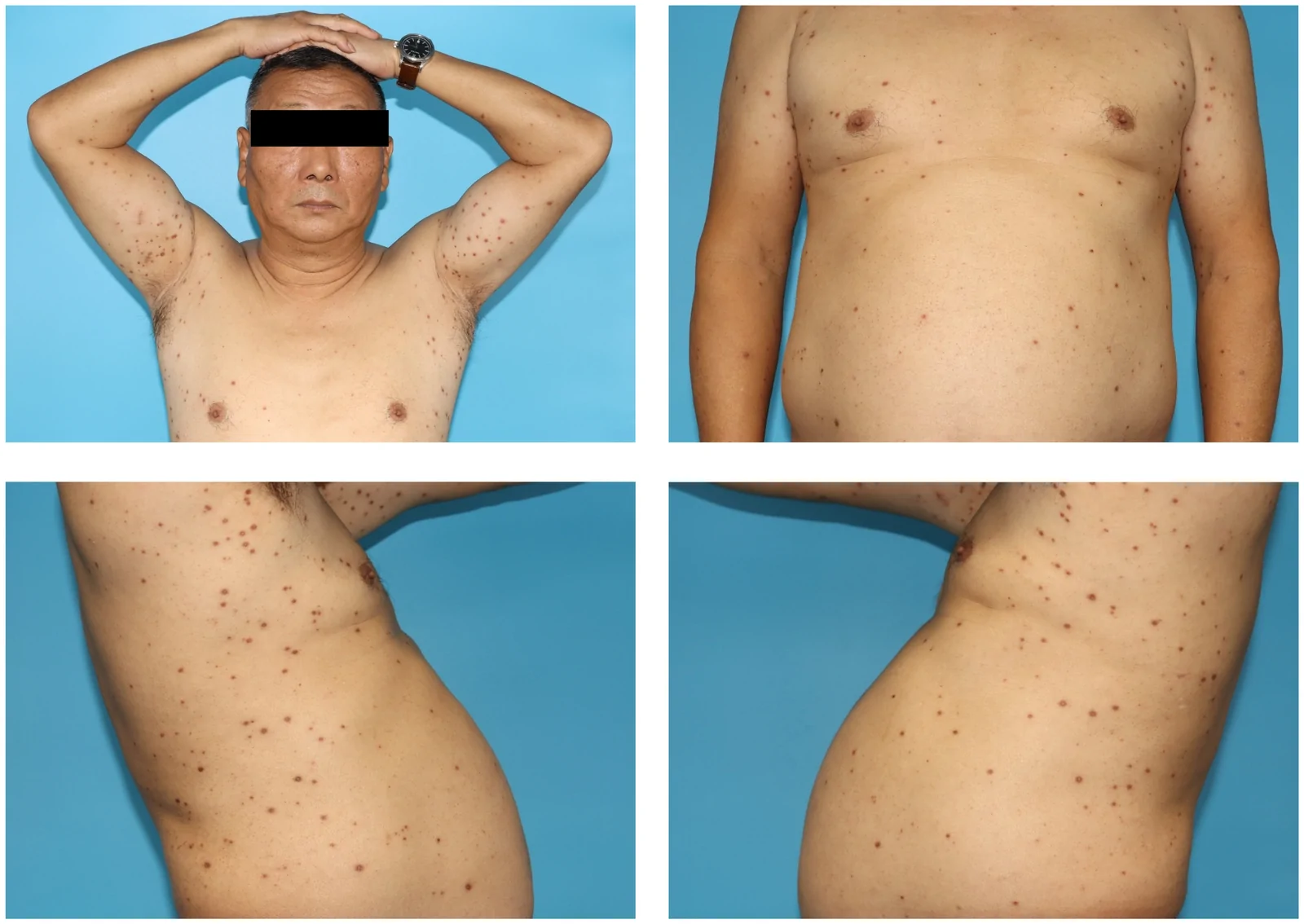
Multiple red papules and plaques on the flanks and inner arms.

**Figure 2:**
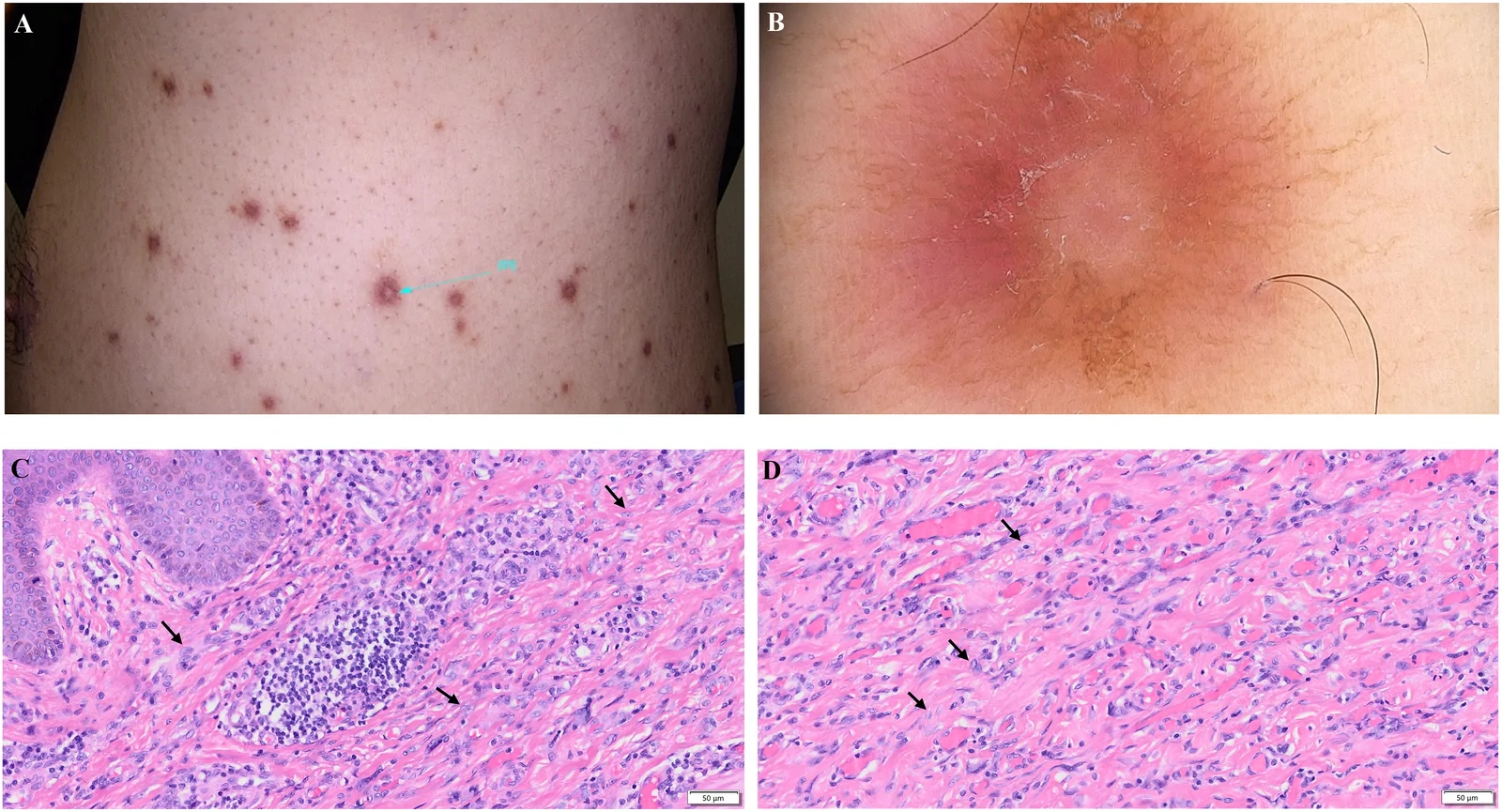
**(A, B)** Dermoscopy revealed a central white structureless area with scant white scales surrounded by a peripheral red structureless area. Focal light-brown atypical reticular structures were present, with hair shafts emerging within the lesion (×20). **(C, D)** Histopathological examination revealed relatively dense infiltration of histiocytes and plasma cells, with a small number of eosinophils, predominantly distributed around superficial to mid-dermal blood vessels. Interstitial infiltration of histiocytes and scattered plasma cells was also observed between collagen bundles. Green arrow indicates the positions selected for dermoscopic photography. Black arrow indicates the histiocytes.

Physical examination revealed multiple dark red to brown papules distributed symmetrically on both sides of the trunk, the medial upper arms, and the medial thighs (**Figure 1**). Dermoscopic examination revealed a central white structureless area with minimal white scaling surrounded by a peripheral red structureless zone; in some lesions, a faint atypical brown reticular pattern was observed, with hair shafts visible within the lesions (**Figure 2A**).

**Figure 3:**
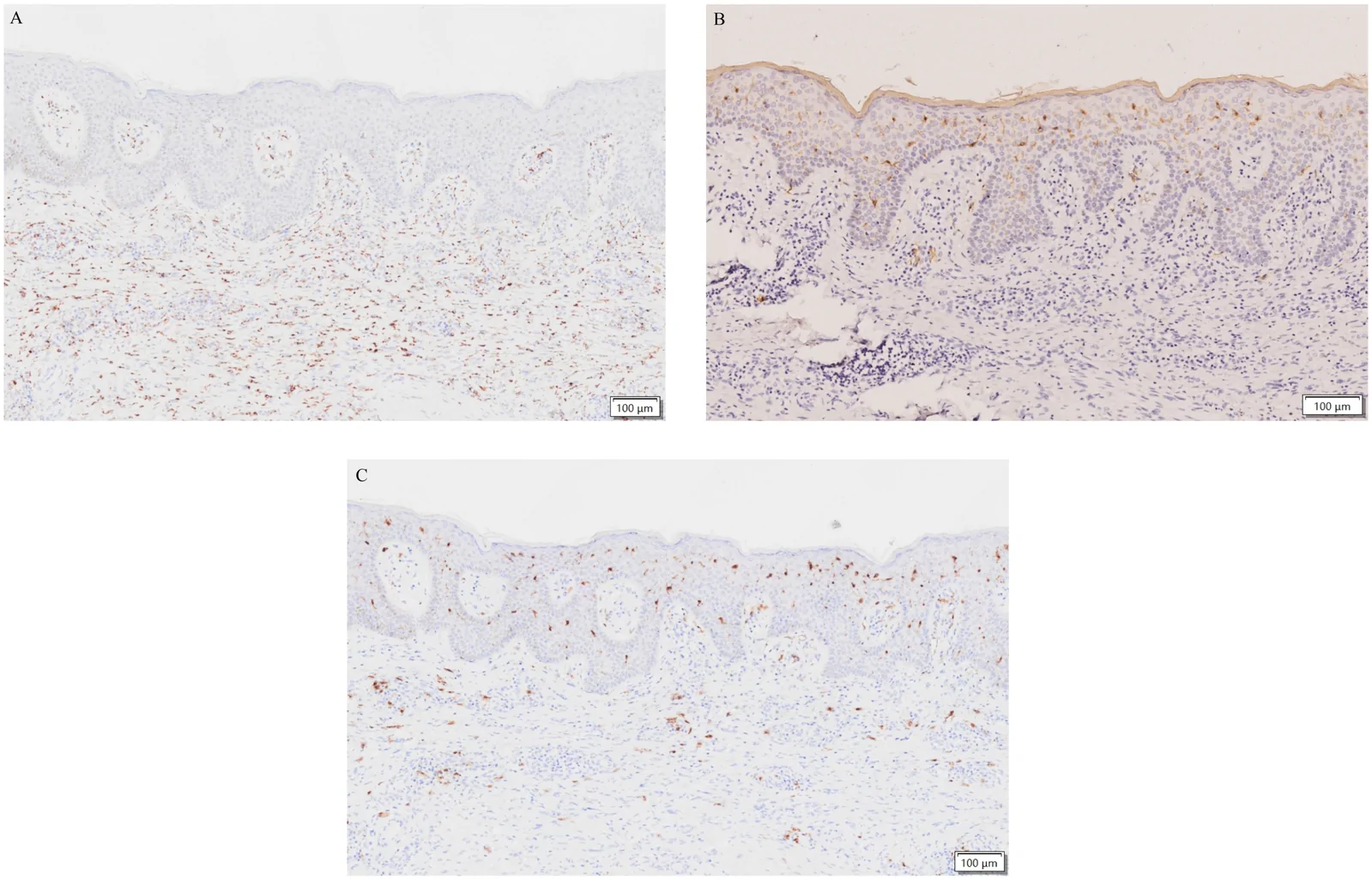
Immunohistochemistry: **(A)** diffuse CD68 positivity, **(B)** CD1a negativity, **(C)** and scattered S-100 positivity.

Histopathological examination of skin biopsy samples, conducted through methods such as Hematoxylin-Eosin staining, revealed that relatively dense infiltrates of histiocytes, lymphoplasmacytic cells, and a small number of eosinophils surrounding superficial to mid-dermal vessels. Similar infiltrates of histiocytes and scattered lymphoplasmacytic cells were also observed between collagen bundles (**Figure 2C**). Immunohistochemical analysis revealed diffuse positivity for CD68, negative staining for CD1a, and scattered positivity for S-100 protein (**Figure 3**). On the basis of these findings, a diagnosis of generalized eruptive histiocytosis (GEH) was established.

**Figure 4:**
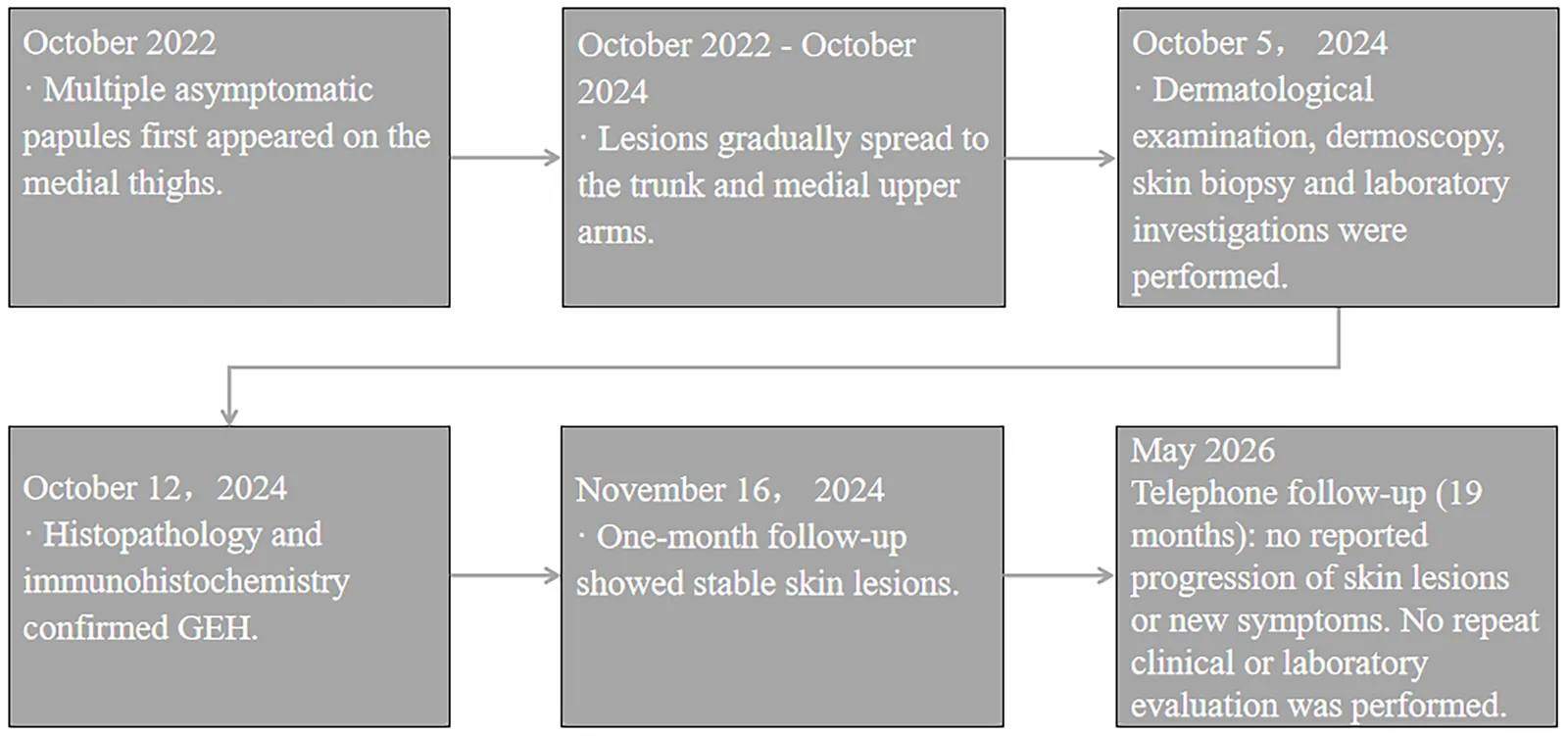
Timeline of the patient’s clinical course, including symptom onset, lesion progression, dermatological evaluation, histopathological diagnosis, and follow-up. This visual summary illustrates the temporal sequence from the initial appearance of skin lesions to the diagnosis of generalized eruptive histiocytosis (GEH) and the subsequent persistent but non-progressive clinical course during the 19-month follow-up.

The biopsy wound was treated with topical neomycin ointment, while no treatment was administered to the remaining lesions. At the 1-month follow-up visit, the patient’s condition remained clinically stable. Laboratory tests, including capillary electrophoresis immunotyping and an automated line immunoblot assay (EUROLineMaster Plus-A, EUROIMMUN, Germany), revealed positive anti-nRNP/Sm antibodies, along with elevated γ-glutamyltransferase, uric acid, and glycocholic acid levels. The patient was followed up via telephone for 19 months until May 2026. During the telephone follow-up, the patient reported no apparent progression or regression of the skin lesions and no new symptoms. No recent clinical or laboratory examinations were performed. The timeline of the patient’s clinical course is shown in **Figure 4**.

## Discussion

In previously reported cases of GEH, immunohistochemical findings typically demonstrate CD68 positivity with negative staining for CD1a and S-100 protein. In contrast, the present case exhibited two relatively uncommon features: scattered S-100 protein expression in lesional histiocytes and serum anti-nRNP/Sm autoantibodies positivity.

In routine pathological practice, immunostaining for S-100 protein is typically employed as an auxiliary diagnostic marker for Langerhans cell histiocytosis (LCH) (4). However, S-100 expression is not disease specific. Studies on GEH have indicated that S-100 positivity alone should neither exclude nor support alternative diagnoses. When the clinical presentation and histopathological features already support a given diagnosis, varying degrees of S-100 expression in lesional histiocytes are not uncommon (5–7). For example, Rosai-Dorfman disease, another non-Langerhans cell histiocytosis, also demonstrates S-100-positive histiocytes, highlighting the non-specific nature of S-100 expression (8). Accordingly, scattered S-100 positivity in this context should be interpreted as a non-specific immunohistochemical finding and considered together with the overall clinicopathological features rather than as evidence supporting an alternative diagnosis or a specific biological process.

In addition to the atypical S-100 expression, laboratory testing in our patient revealed serum anti-nRNP/Sm autoantibodies. Anti-nRNP/Sm antibodies belong to the spectrum of extractable nuclear antigen (ENA) antibodies and are mainly associated with systemic autoimmune disorders, particularly mixed connective tissue disease (MCTD) and systemic lupus erythematosus (SLE) (9, 10). Nevertheless, the presence of anti-nRNP/Sm antibodies alone is insufficient to establish a diagnosis of MCTD or SLE, and the diagnosis of these diseases requires observation of clinical manifestations and longitudinal follow-up (10). To date, no systematic studies have demonstrated a definitive association between GEH and persistent anti-nRNP/Sm positivity or specific connective tissue diseases. In our patient, no clinical evidence of systemic autoimmune disease was identified during follow-up, suggesting that anti-nRNP/Sm positivity may represent an underlying immunological abnormality rather than an established autoimmune disease.

The coexistence of scattered S-100-positive histiocytes and anti-nRNP/Sm autoantibodies raises the possibility of a broader dysregulation of immune homeostasis in this patient. Histiocytes are not only involved in tissue remodeling but also participate in innate immune regulation and antigen presentation. Therefore, altered histiocytic activation or differentiation may represent a potential interface between cutaneous histiocytic proliferation and systemic immune abnormalities. Nevertheless, whether these two findings share a common pathogenic mechanism or represent independent immunological events remains unclear and requires further investigation.

The differential diagnosis includes the following conditions.

1. Langerhans cell histiocytosis (LCH): LCH can occur at all ages but is most prevalent in children and is often associated with systemic or organ involvement (11). Histopathologically, it is characterized by proliferating Langerhans cells with grooved nuclei and abundant eosinophilic cytoplasm. Immunohistochemically, these cells are typically positive for CD1a, Langerin/CD207, and the S-100 protein, while CD68 expression is usually negative or weak (12). Ultrastructural examination revealed characteristic Birbeck granules with rod- or tennis racket–like morphology. In the present case, the immunostaining results for CD1a were unequivocally negative, arguing against LCH.
2. Adult xanthogranuloma: Histopathology typically reveals foamy histiocytes and characteristic Touton giant cells with a wreath-like nuclear arrangement (13). Touton giant cells were not observed in this case, making this diagnosis unlikely.
3. Xanthoma disseminatum (XD): XD predominantly affects males and commonly occurs in children and young adults, frequently involving the skin and mucous membranes. Lesions present as symmetrically distributed erythematous to yellow–brown papules and nodules on the scalp, face, trunk, and proximal extremities, often forming confluent plaques. Histologically, spindle-shaped histiocytes and vacuolated lipid-laden cells are observed (14). These features were absent in the present case, excluding XD.

The etiology of GEH remains unclear. Some authors have suggested that GEH may represent an early stage within a spectrum of macrophage-related disorders, such as juvenile xanthogranuloma (JXG), XD, and progressive nodular histiocytosis (PNH) (15). GEH is generally regarded as a rare, benign, and self-limited histiocytic disorder, with most cases confined to the skin and lesions resolving spontaneously over weeks to years, leading to a favorable overall prognosis. However, when cutaneous histiocytosis presents with generalized eruptions accompanied by risk-organ involvement, clinicians should be highly alert to the possibility of concomitant myeloid neoplasms and conduct further comprehensive examinations. To date, only a limited number of studies have reported GEH associated with systemic involvement or hematologic disorders (16). For example, Ziegler et al. reported an adult case of GEH occurring in association with chronic eosinophilic leukemia harboring the FIP1L1–PDGFRA fusion gene (17). Dobrosavljevic et al. suggested that in preschool children, the evolution of GEH lesions towards a xanthomatous appearance should prompt consideration of concomitant diabetes insipidus, whereas hematologic follow-up is recommended in adult patients to exclude underlying hematologic abnormalities (18). Therefore, despite its generally favorable prognosis, appropriate follow-up strategies should be tailored according to patient age, lesion evolution, and the presence of systemic symptoms. Various therapeutic approaches—including phototherapy, isotretinoin, methotrexate, hydroxychloroquine, thalidomide, cyclophosphamide, and systemic corticosteroids—have been reported, with inconsistent efficacy (19), and no standardized treatment regimen has been established. In the present case, no systemic therapy was administered. At the 1-month follow-up, the lesions had not significantly progressed, and the patient remained in good general condition. During a subsequent 19-month telephone follow-up, the patient reported no apparent progression or regression of the lesions and no new symptoms; however, objective evaluation was limited by the lack of recent clinical and laboratory examinations.

This case has several limitations. Follow-up was conducted by telephone without repeat physical examination or laboratory reassessment. Therefore, the follow-up findings should be interpreted with caution when assessing possible systemic autoimmune involvement.

## Conclusion

In conclusion, the diagnosis of GEH relies on a comprehensive approach that integrates clinical presentation, histopathological findings, and the immunophenotypic findings. While the histiocytic infiltrate in this patient exhibited a classic CD68+/CD1a- pattern, the combination of scattered S-100 positivity and concurrent serum anti-nRNP/Sm antibodies represents an uncommon immunophenotypic finding. Scattered S-100 positivity should be interpreted as a non-specific immunohistochemical finding rather than evidence of Langerhans cell differentiation, and should be interpreted in conjunction with the overall clinicopathological features. The presence of anti-nRNP/Sm antibodies should be interpreted cautiously in the absence of compatible clinical manifestations, as isolated seropositivity alone is insufficient to establish an underlying connective tissue disease. This case highlights the importance of comprehensive clinicopathological evaluation and careful interpretation of atypical immunohistochemical and serological findings in patients with GEH. Further well-characterized case reports and longitudinal clinical studies are needed to better define the clinical significance of these findings.

## Data Availability

The original contributions presented in the study are included in the article/supplementary material. Further inquiries can be directed to the corresponding authors.
